# Supplementary material for: Serological evidence and factors associated to liver damage in malaria-typhoid infected patients consulting in two health facilities, Yaoundé-Cameroon
Source: PLoS One. 2025 May 23;20(5):e0319547. doi: 10.1371/journal.pone.0319547 (PMC12101696; doi:10.1371/journal.pone.0319547)
Supplement: S1 File — (DOCX) [file pone.0319547.s001.docx]

**CONSENT FORM**

**I, the undersigned, Mr/Mrs/Miss……………………………………………………………..,**

**have been invited to participate in the research study entitled: “Assessment of the impact of malaria, typhoid or malaria-typhoid co-infection on liver function markers among patients with febrile fever consulting at the CMA of Obili** **and the** **Mvog-Besti Dominican Hospital Center, Yaoundé, Cameroon, Yaoundé-Cameroon”.**

**• I fully understood the information given to me based on this study.**

**• I clearly understood the goals and objectives of this study.**

**• I received answers to all my questions asked.**

**• I understood that I have the free choice to accept or decline my participation in this study. • My consent to take part in this study does not give the investigator a total right over me, because I know my right as per the law.**

**I accept at my free will to take part in this study based on the information provided by the investigator of this study to:**

** Answer the research questions**

** Communicate my medical reports**

** Give 5 ml of my blood**

**Done at………….……………, on…..…………………….**

**Signature of participant**

**STRUCTURED-QUESTIONNAIRE**

**Project title: ““Assessment of the impact of malaria, typhoid or malaria-typhoid co-infection on liver function markers among patients with febrile fever consulting at the CMA of Obili and the Mvog-Besti Dominican Hospital Center, Yaoundé, Cameroon, Yaoundé-Cameroon””.**

**Questionnaire title: The Burden of Malaria and typhoid fever at CMA of Obili in Yaoundé-Cameroon**

**Participant Code...……… Date…………………**

**Section one: Socio-demographic status**

**1. Age:** [0-5] ; ]5-20] ; ]20-40] ; ]40-60] ; > 60

**2. Gender:** (1) Male (2) Female

**3. Marital status:** (1) Married (2) Single (3) Widow (4) Widower

**4. Profession:** (1) Civil servant; (2) Employee; (3) Self-employment;

(5) Others (specify……………………………….)

**5. Level of Education:** (1) Never been to school; (2) Primary school; (3) Secondary school (4) University

**6. House type:** (1) Mud block (2) Cement block (3) Wood

**7. Number of people in the household: ………………………..**

**8. Is there a ceiling?** (1) Yes (2) No

**9. If yes, type of ceiling:** (1) Plywood ; (2) Zinc ; (3) Bamboo ; (4) Others

(specify…………………………)

**10. Is there space at the gable ends of the wall:** (1) Yes ; (2) No

**11. Type of toilet:** (1) Pit latrine (2) Water system

**12. Do you often stay out of your home late into the nights?** (1) Yes ; (2) No

**13. What is your monthly income level: …………………………………**

**14. What is the source of your running water?** (1) Well ; (2) Tap water ; (3) Spring water ; (4) Borehole water

**15. What is the source of your drinking water?** (1) Mineral water ; (2) Tap water ; (3) Spring water ; (4) Borehole

**Section Two: Prevention and management of malaria**

**1. Do windows have mosquito nets?** (1) Yes ; (2) No

**2. Is there standing water within 20 meters around the house:** (1) Yes ; (2) No

**3. Are there bushes around the house:** (1) Yes ; (2) No

**4. Are there crops around the house:** (1) Yes ; (2) No

**5. If yes, what type:** (1) Plantain ; (2) Cocoa ; (3) Coffee ; (4) Cocoyam

(5) Others (specify………………………..)

**6. Do you have a mosquito net?** (1) Yes ; (2) No

**7. If yes, how frequent do you use it:** (1) everyday (2) Some days (3) Don’t use

**8. What is the state of the net:** (1) Old (2) Fairly use (3) New

**9. Dose the net have holes:** (1) Yes (2) No

**10. Have you ever seen a dead insect on your net:** (1) Yes (2) No

**11. Have you ever seen a dead mosquito on your net:** (1) Yes (2) No

**12. Where did you obtain the net:** (1) Market (2) Health center (3) Community health campaign

**13. When did you obtain the net:** (1) A month ago (2) 6 months ago (3) 1 year ago

(4) Others (specify…………………….)

**14. Do you use insecticide mosquito spray:** (1) Yes (2) No

**15. If yes, how frequent do you use it: (**1) everyday (2) Some days (3) Don’t use

**16. How do you store water in the house:** (1) Open containers (2) Close containers?

(3) Others (specify…………………….)

**17. Do you take malaria drugs as prophylaxis?** (1) Yes (2) No

**18. If yes, which class of drug do you take:** (1) Mefloquine (2) Sulfadoxine - Pyrimethamine

(3) Primaquine (4) Chloroquine

**19. Have you ever had malaria:** (1) Yes (2) No

**20. If yes, when did you last have malaria:** (1) This month (2) Five month ago

(3) One year ago

**21. Where do you take treatment for malaria:** (1) Health center (2) Drug store

(3) Herbalist (4) Auto medication (5) Others (specify………………)

**(b) Which drug did you use**: (1) Athermeter (2) Quinine (3) Don’t know

(4) Others (specify……………….)

**22. After how many days did you get to the hospital after the first clinical symptoms:** (1) Less than 3 days; (2) between 3 and 7 days; (3) more than 7 days

**Section Three: Prevention and management of typhoid**

**1. What is the source of the house whole drinking water:** (1) Mineral water (2) Tap water (3) Borehole water (4) Spring water

**2. Do you treat the water before drinking?** (1) Yes (2) No

**3. If yes, what is the method of treatment:** (1) Filtration; (2) Boiling (3) Others (specify………………………..)

**4. Do you wash vegetables, food stuffs and fruits before cooking or eating?** (1) Yes (2) No

**5. Do you cover left over food after eating?** (1) Yes (2) No

**6. Is the compound infested with flies?** (1) Yes (2) No

**7. Are there garbage dumps around the house?** (1) Yes (2) No

**8. What is your toilet system:** (1) Pit latrine (2) Water system

**9. Do you wash your hands before and after meal?** (1) Yes (2) No

**10. Do you practice proper hand hygiene after using the toilet?** (1) Yes (2) No

**11. Have you ever had typhoid before?** (1) Yes (2) No

**12. If yes, when did you have typhoid the last time?** (1) A month ago (2) 6 months ago (3) 1 year ago (4) Others (specify…………………….)

**13. After how many days did you get to the hospital after the first clinical symptoms:** (1) Less than 3 days; (2) between 3 and 7 days; (3) more than 7 days

**14. Do you take prophylaxis against typhoid?** (1) Yes (2) No

**15. If yes, what class of drug do you take: (**1) Vivotif (2) Typhimvi (3) Typhoid vaccine (4) Others (specify………………………)

**16. Where do you take treatment for malaria?** (1) Health center (2) Drug store (3) Herbalist (4) Auto medication (5) Others (specify………………………)

**17. What medication do you take to treat typhoid:** (1) Ceftriaxone (2) Ciprofloxacin

(3) Erythromycin (4) Levofloxacin (5) Norfloxacin

**Thank you for your time and consideration!!!!!!!!!**
