# Supplementary material for: Serological evidence and factors associated to liver damage in malaria-typhoid infected patients consulting in two health facilities, Yaoundé-Cameroon
Source: PLoS One. 2025 May 23;20(5):e0319547. doi: 10.1371/journal.pone.0319547 (PMC12101696; doi:10.1371/journal.pone.0319547)
Supplement: S2 File — (DOCX) [file pone.0319547.s002.docx]

**Supplementary Tables**

**Full title: Serological evidence and factors associated to liver damage in malaria-typhoid infected patients consulting in two health facilities, Yaoundé-Cameroon**

**Short title: Liver damage in malaria-tyhoid infected patients, Yaoundé-Cameroon**

**S1_Table: Socio-demographic characteristics of febrile patients attending CMA of Obili and CHD of Mvogt-betsi and enrolled in this study.**

| **Socio-demographic characteristics** | **Categories** | **Frequency (n)** | **Percentage (%)** |
| --- | --- | --- | --- |
| **Gender** | Female | 232 | 66.3 |
|  | Male | 118 | 33.7 |
|  | **Total** | **350** | **100.0** |
| **Age group** | [0-5] | 19 | 5.4 |
|  | ] 5-20] | 68 | 19.4 |
|  | ] 20-40] | 171 | 48.9 |
|  | ] 40-60] | 51 | 14.6 |
|  | >60 | 41 | 11.7 |
|  | **Total** | **350** | **100.00** |
| **Marital status** | Single | 202 | 57.7 |
|  | Married | 127 | 36.3 |
|  | Divorced | 6 | 1.7 |
|  | Widow(er) | 15 | 4.3 |
|  | **Total** | **350** | **100.0** |
| **Level of education** | Not-schooled | 9 | 2.6 |
|  | Primary | 39 | 11.1 |
|  | Secondary | 103 | 29.4 |
|  | University | 199 | 56.9 |
|  | **Total** | **350** | **100.0** |
| **Profession** | Civil servant | 47 | 13.4 |
|  | Employee | 70 | 20.0 |
|  | Self-employment | 233 | 66.6 |
|  | **Total** | **350** | **100.0** |
| **Type of house** | Cement block | 310 | 88.6 |
|  | Wood | 9 | 2.6 |
|  | Mud block | 31 | 8.8 |
|  | **Total** | **350** | **100.0** |
| **Type of toilet** | Pit latrine | 114 | 32.6 |
|  | Water system | 236 | 67.4 |
|  | **Total** | **350** | **100.0** |
| **Source of running water** | Borehole | 165 | 47.1 |
|  | Well | 81 | 23.1 |
|  | Spring | 26 | 7.5 |
|  | Tap water | 78 | 22.3 |
|  | **Total** | **350** | **100.0** |
| **Source of drinking water** | Mineral | 54 | 15.5 |
|  | Borehole | 211 | 60.3 |
|  | Spring | 18 | 5.1 |
|  | Tap | 67 | 19.1 |
|  | **Total** | **350** | **100.0** |

**S2_Table: Clinical symptoms experienced by febrile patients enrolled in the study**

| **Clinical symptoms** | **Categories** | **Frequency (n)** | **Percentage (%)** |
| --- | --- | --- | --- |
| **Fever** | Yes | 292 | 83.4 |
|  | No | 58 | 16.6 |
|  | **Total** | **350** | **100.0** |
| **Headaches** | Yes | 167 | 47.7 |
|  | No | 183 | 52.3 |
|  | **Total** | **350** | **100.0** |
| **Chills** | Yes | 115 | 32.9 |
|  | No | 235 | 67.1 |
|  | **Total** | **350** | **100.0** |
| **Asthenia** | Yes | 174 | 49.7 |
|  | No | 176 | 50.3 |
|  | **Total** | **350** | **100.0** |
| **Muscles aches** | Yes | 114 | 32.6 |
|  | No | 236 | 67.4 |
|  | **Total** | **350** | **100.0** |
| **Abdominal pain** | Yes | 116 | 33.1 |
|  | No | 234 | 66.9 |
|  | **Total** | **350** | **100.0** |
| **Diarrhea** | Yes | 46 | 13.1 |
|  | No | 304 | 86.9 |
|  | **Total** | **350** | **100.0** |
| **Vomiting** | Yes | 77 | 22.0 |
|  | No | 273 | 78.0 |
|  | **Total** | **350** | **100.0** |

**S3_Table: Attitudes of feverish patients towards adherence to preventive measures for malaria and typhoid fever**

| **Preventive measures** | **Categories** | **Frequency (n)** | **Percentage (%)** |
| --- | --- | --- | --- |
| **Stay out late at night** | Yes | 99 | 28.3 |
|  | No | 251 | 71.7 |
|  | **Total** | **350** | **100.0** |
| **Standing water near the house** | Yes | 60 | 17.1 |
|  | No | 290 | 82.9 |
|  | **Total** | **350** | **100.0** |
| **Crops near the house** | Yes | 54 | 15.4 |
|  | No | 296 | 84.6 |
|  | **Total** | **350** | **100.0** |
| **Bushes near the house** | Yes | 113 | 32.3 |
|  | No | 237 | 67.7 |
|  | **Total** | **350** | **100.0** |
| **Windows with nets** | Yes | 146 | 41.7 |
|  | No | 204 | 58.3 |
|  | **Total** | **350** | **100.0** |
| **Use of mosquito nets** | Regular | 218 | 62.3 |
|  | Irregular | 55 | 15.7 |
|  | Don’t use | 77 | 22.0 |
|  | **Total** | **350** | **100.0** |
| **Use of insecticide mosquito spray** | Regular | 49 | 14.0 |
|  | Irregular | 46 | 13.1 |
|  | Don’t use | 255 | 72.9 |
|  | **Total** | **350** | **100.0** |
| **House with ceiling** | Yes | 319 | 91.1 |
|  | No | 31 | 8.9 |
|  | **Total** | **350** | **100.0** |
| **Malaria prophylaxis** | Yes | 25 | 7.1 |
|  | No | 325 | 92.9 |
|  | **Total** | **350** | **100.0** |
| **purify water before drinking** | Yes | 78 | 22.3 |
|  | No | 272 | 77.7 |
|  | **Total** | **350** | **100.0** |
| **washing fruits and vegetables before consumption** | Yes | 322 | 92.0 |
|  | No | 28 | 8.0 |
|  | **Total** | **350** | **100.0** |
| **hand washing before meals** | Yes | 310 | 88.6 |
|  | No | 40 | 11.4 |
|  | **Total** | **350** | **100.0** |
| **garbage dumps near the house** | Yes | 63 | 18.0 |
|  | No | 287 | 82.0 |
|  | **Total** | **350** | **100.0** |

**S4_Table: Attitudes of feverish patients towards management and treatment of malaria infection**

| **Management and treatment** | **Categories** | **Frequency (n)** | **Percentage (%)** |
| --- | --- | --- | --- |
| **Time taken before consultation** | < 3 days | 155 | 44.3 |
|  | [3-7] days | 169 | 48.3 |
|  | ˃ 7 days | 26 | 7.4 |
|  | **Total** | **350** | **100.0** |
| **Place of malaria treatment** | Health center | 213 | 60.9 |
|  | Self-medication | 112 | 32.0 |
|  | Herbalism | 25 | 7.1 |
|  | **Total** | **350** | **100.0** |
| **Drugs used for malaria treatment** | ACT | 257 | 73.4 |
|  | Chloroquine | 66 | 18.9 |
|  | Traditional medicine | 27 | 7.7 |
|  | **Total** | **350** | **100.0** |
| **Place of typhoid fever treatment** | Health center | 265 | 75.7 |
|  | Self-medication | 14 | 4.0 |
|  | Herbalism | 71 | 20.3 |
|  | **Total** | **350** | **100.0** |
| **Drugs used for malaria treatment** | Ceftriaxone | 174 | 49.7 |
|  | Ciprofloxacin | 105 | 30.0 |
|  | Traditional medicine | 71 | 20.3 |
|  | **Total** | **350** | **100.0** |

*ACT: Artemisinin-Based Combination Therapy*
