# Supplementary material for: Serological evidence and factors associated to liver damage in malaria-typhoid infected patients consulting in two health facilities, Yaoundé-Cameroon
Source: PLoS One. 2025 May 23;20(5):e0319547. doi: 10.1371/journal.pone.0319547 (PMC12101696; doi:10.1371/journal.pone.0319547)
Supplement: Attachment — Submitted filename: REVIEWERS RECOMMENDATION for plosONE.docx [file pone.0319547.s004.docx]

**REVIEWERS RECOMMENDATION**

**Serological evidence and factors associated to liver damage in malaria-typhoid infected patients consulting in two health facilities, Yaoundé-Cameroon.**

This study focused on an often-neglected area of the impact of malaria and typhoid on the liver function of infected patients in two clinics in Yaoundé-Cameroon. The authors identify risk factors for infections and liver injury by using questionnaires, microscopy (malaria), Widal tests (typhoid), and liver enzyme assays. Key findings include higher infection rates in males, environmental risk factors (e.g., standing water), and co-infection/delayed consultation as significant predictors of liver damage. This is good research that addresses a critical gap in understanding liver injury in Africa. However, there are concerns,

- The abstract (lines 47-48) says, “Men (58/118 and 44/118) were more affected than 48 women (71/232 and 62/232) for both malaria and typhoid”. I believe using percentage here will communicate your result better than the proportion.
- The Widal test used for determining typhoid has been criticized for having low sensitivity and specificity. Can another confirmatory test be used to establish typhoid in the patients, or do you acknowledge this limitation in your discussion?
- The study needs a control group of non-febrile individuals to compare the baseline liver enzyme levels in the general population.
- There is a need to consider other cofounders, like previous or active viral infections such as hepatitis, that can further influence liver enzyme levels. Also, there is a need to analyze the potential confounding effect of drug-induced liver injury versus pathogen-induced liver damage. Statistically, you can also perform multivariate analysis to adjust for confounders.
- In the method section, there is no clear description of how consent from underage patients was obtained.
- While the Roussel Uclaf Causality Assessment Method (RUCAM) is an acceptable tool, it is typically used for drug-induced liver injury. Its applicability to infectious diseases should be justified.
- Some thresholds used in the method section were not clearly defined. For example, lines 205-206, “*In our laboratory, these reference values in humans are: from 10 to 42 UI/L, from 8 to 39 UI/L, and from 40 to 129 UI/L respectively for ALT, AST, and ALP*.” How does your reference value compare to those used in other countries, and how did you arrive at your reference values? Also, the threshold for classification of parasite density (low: <500, medium: 500–2500, high: >2500 parasites/μL) should be backed with references
- The figures are not up to standard. Please improve the quality of your graphs.

There are a lot of grammatical errors on the document, and I will recommend a thorough revision of the manuscript. Some of the ones spotted are listed below:

- Line 38 - "risk factors associated to" should be "risk factor associated with".
- Line 40 - "During 8 months" should be "Over 8 months"
- Line 43 - "Liver damage was assessed employing" should be “Liver damage was assessed using"
- Line 54 - "Liver enzyme activities, reflecting liver damages" should be "Liver enzyme activity, reflecting liver damage"
- Line 56 - "should be considered during the patient's treatment." should be "should be considered during patient treatment."

In the discussion, there are several sentences that needs to be splitted into clearer sentences. For example; “*However, current diagnostic procedures and management approaches do not consider evaluating liver damage in infected individuals, even though their causative organisms, Plasmodium species for malaria and Salmonella serotypes for typhoid fever, have a necessary hepatic phase in their respective pathogenic mechanisms, which is not without*

*adverse consequences on the liver, and this can increase the morbidity and mortality as a result of complications arising from liver injuries*.”
